# Customs to Be Condemned

**Published:** 1921-05-07

**Authors:** 


### THE HEALTH OF A CHILD.

### Customs to be Condemned.

The future of our race depends upon the child, and it is a welcome sign that the public is gradually realising the fact. Unfortunately the best methods by which to raise the average health standard in children have not even yet crystallised into a generally accepted scheme. Appreciating that a host of well-meaning people are daily engaged in welfare work, whose efforts suffer nevertheless from the influence of established popular ideas and certain hard-dying fads of professional practice, one is prompted to outline a few of the discrepancies.

### The Influence of Tradition.

To address, in the first place, the professional worker, let us impress the fact that race welfare does not depend upon the medical treatment of sick children but upon actual prevention of that sickness. Prevention is possible only through the intelligent co-operation of the public, and parents can only co-operate when they have an intelligent idea of what is expected of them. It is useless to teach them rule-of-thumb methods. They must be brought to understand the true reason for many things they are urged to do. Otherwise it is hopeless to expect the unusual circumstance to be intelligently met. But one of the great difficulties lies in the influence of tradition. In no department of the whole field of medicine does this factor predominate so strongly as in child welfare work. And it is not enough to say that a procedure is wrong. Sound reasons must be advanced which will appeal to the common sense of the average individual.

Let us come, therefore, down to first principles. In a practical sense we must always regard the child simply as one of an animal species and subject to the natural laws which animals observe. If it is normal at birth, its future largely depends on the care and nutrition which it receives. But its career does not begin only at birth. Throughout the whole of gestation it is dependent on the mother, and pre-natal care is an absolute necessity, not necessarily for the prevention of malpositions, etc., but for adequate nutrition and development of an intrinsically normal fœtus. As a matter of fact, the idea that a large percentage of deliveries require artificial interference is, in itself, fallacious, for in European countries records show that well over 90 per cent. of births occur without any instrumental aid. Therefore let it be urged, when the risks in all instrumentation are admitted, that among many forceps injuries, not a few might be avoided by a policy of intelligent and watchful waiting. In short, the child must cease to be ever considered a relative by-product in obstetrics. The circumstances of its birth exert an all-powerful influence upon its ultimate health.

### Customs and Facts.

That a baby must be bathed directly after birth is far from true. Infants rapidly lose heat, and should the warmth of the room or water be insufficient, the risks attendant upon a subnormal temperature (bronchitis, etc.) are at once incurred. Acute, even minutely pustular, dermatitis is readily caused by well-meant efforts to remove the vernix caseosa, and the condition is readily exaggerated by the use of a improperly neutralised soap. Another common fallacy, and one causing frequent harm, is the swabbing of the mouths of new-born infants. The gauze so frequently used always strips more or less of the delicate epithelium from the mucous membrane, and is the true cause of some stomatitis cases. The stump of the cord does not require an antiseptic dusting powder. It will mummify and become detached more quickly, and certainly with less danger of infection, if allowed to remain in contact with the air under a simple thickness of gauze. In connection with the belief that exactly daily stools are necessary in a breast-fed child it is noteworthy that Professor Ramsay in a long series of cases in the University of Minnesota Hospital, none of which had any cathartic drug, finds the normal average distinctly higher. In any case, in young infants the normal reflex will occur when sufficient residue has collected, and it is probable that the classical dose of castor oil on the third day is responsible for much of the digestive disturbance often attributed to other causes. The observer already quoted finds that infantile stools, if examined microscopically after a dose of castor oil, usually contain blood. If the doses have been repeated frequently they always do. Castor oil certainly cannot claim the "soothing elements" it is often reputed to possess. Another diagnostic point to be realised is that normal "teething" is not attended by such symptoms as fever, diarrhœa and convulsions. It may do as a satisfying explanation, but on such occasions there is undoubtedly another cause to be sought, though it be only temporary in nature. The eruption of the teeth is a physiological process like the growth of any other organ.

### Physiological Growth.

And mention of normal growth brings one to the question of watching the infant's early developments. All are so familiar with the typical weight chart and the classical dates and figures that repetition of their details and meaning need not detain us. But in general welfare work it is only in favourable circumstances that these records are accurately kept. Therefore one must look for outstanding points, particularly such things as the parents are likely themselves to have noted, and therefore be able to help in providing the evidence. In six months and a year respectively a child should about double and treble its weight at birth. If its height at birth is about 20 inches it should measure about 28 inches at the end of the first year, and another four at the end of the second. The milk teeth are usually complete shortly after two and a-half years, and in any event at the end of the third. One mentions these simple points because there is an extraordinary amount of misunderstanding among the public upon normal growth figures, and, well intentioned though they are, the complicated records presented to them by some clinics are really not one-quarter understood. It would be far preferable to popularise some simple standards.

In the effort to make a perfectly satisfactory child " grow " an enormous number of cases of true overfeeding occur. The conditions of modern civilisation have cumulatively tended to multiply the number of children with relatively unstable nervous systems, and in these no greater error than overfeeding can be made. The instinctive idea is that perhaps more nourishment, and haphazard " feeding up " will improve the condition, and yet there is no constitutional disability in children for which more can be accomplished by intelligent and careful gradation of the diet. But details are beyond the scope of this note. Here and elsewhere we have given simple outline indications, and have tried to illustrate the urgent educative principle involved.

